# London's New Pay Hospital

**Published:** 1903-01-17

**Authors:** 


### LONDON'S NEW PAY HOSPITAL.

### OPENING BY THE DUKE OF NORTHUMBERLAND.

A number of ladies and gentlemen assembled on Friday afternoon (9th inst.), when the new Pay Hospital in Fitzroy Square was opened.

The chair was taken by his Grace the Duke of Northumberland, who was supported by Sir Henry Burdett, Sir William Church, President of the Royal College of Physicians, the Bishop of Southwark, and Mr. Arthur B. Burdett, hon. secretary. There was a large and representative attendance of governors and members of the medical profession. Letters of apology were read from the Bishop of Winchester (the new Archbishop of Canterbury), the Earl of Aberdeen, and Mr. A. F. Walter.

#### The Dedication.

The president, after welcoming those who were present, asked the Bishop of Southwark to dedicate the new hospital. The service of dedication, said by the Bishop, consisted of the Lord's Prayer and a few Collects, concluding with the words, "In the name of the Father, the Son, and the Holy Ghost we dedicate this hospital to the service of God in man and man in God."

#### The Treasurer's Report.

Sir Henry Burdett, in presenting the treasurer's statement, said he greatly regretted that, owing to a misunderstanding about the date of the meeting, Mr. Frederick Cox, the treasurer, was unable to be present at the commencement; he had desired the speaker to express his sincere regret that the mistake should have occurred. Mr. Cox was one whose work in connection with the hospital had been devotedly carried on during the 22 years of its existence. He had faithfully visited it every month and personally attended to its expenditure. Everyone who had known the hospital during that time must appreciate Mr. Cox's services, and those still to come would reap the benefit of his devotion. In another respect, also, Mr. Cox's name was one of special interest; he was one of the few who survived, out of the number of those who, a quarter of a century ago, were instrumental in founding this hospital. The Duke of Northumberland, Lord Aberdeen, Mr. F. D. Mocatta, Mr. Albert G. Sandeman, Mr. Cox, and himself were the Governors who had been present when the late Bishop of Winchester dedicated the first hospital. Mr. A. F. Walter had written to express his regret at being absent, because his father took a very deep interest in the movement. About the year 1870 he spoke on the subject at a meeting at the Great Ormond Street Hospital, and he was one of the first who put forward the idea of paying patients. Coming to the question of finance, Sir Henry explained the position. The hospital, he said, had been run entirely as a pay hospital from the outset, and it had always paid its way. When it was started, the idea was to charge enough to allow of a sinking fund, in view of the eventuality of rebuilding. The amount charged at the instance of the late Sir Richard Quain was reduced to £3 3s. a week for those who could not afford more, so it was not found possible to put by for a sinking fund on these terms; hence the present request for £10,000 for rebuilding. After 22 years' experience it was wise to point out that accommodation like that at Fitzroy House could not be obtained anywhere else at so little as £4 4s. a week, and it had been decided to adopt the policy of not accepting patients at a less rate in the future. These terms were inclusive, but did not include the doctor's fee.

#### A Right Principle.

He mentioned this fact because those who were responsible had had serious doubts as to whether they ought to go on with the work or not. They had proved that the principle was a right one, and they might therefore be said to have fulfilled their mission. But the representations of the doctors, nearly 1,500 of whom had had patients in the hospital, encouraged them to go on. Members of the medical profession declared that it was almost the only place where they could send their patients with the knowledge that the charges were reasonable and inclusive. There was therefore a public need for its continuance, as well as a strong desire on the part of the medical profession, and the Committee had decided to raise, for purposes of extension, the sum of £10,000. Towards this amount over £2,000 had already been received; there remained, therefore, a balance of some £8,000 to be secured.

#### Wanted: 200 Intelligent Heads of Families.

Now, the speaker continued, there was an opportunity for the head of every household to secure priority of admission for himself and members of his family to this hospital by subscribing a sum of 50 guineas. If there were 200 intelligent heads of households, this was their opportunity. Twenty guineas would secure the privilege of priority of admission for a bachelor or spinster. As soon, however, as the necessary sum had been subscribed these privileges could not be secured. The hospital, it must be remembered, was entirely self-supporting, and on its present basis the committee had no doubt it would continue to be so. The excellence of everything now provided should preclude the necessity of facing, say for another 20 years, the question of rebuilding; however, in the event of this becoming necessary, he was able to state that the terms, as now fixed, were such as to allow of a sinking fund. The hospital henceforward should therefore be self-supporting for all time.

#### A Resolution and a Retrospect.

A resolution was next moved by the president and seconded by Sir William Church, Bart., K.C.B., President of the Royal College of Physicians, to the following effect: "That 22 years' experience of the working of the Home

Hospitals Association proves the utility of a pay hospital for patients of moderate means, conducted upon the system established at Fitzroy House in 1881; and that this success should ensure the immediate provision by the public of the £10,000 required to defray the cost of rebuilding the Pay Hospital, 16, 17, and 18 Fitzroy Square."

The Duke of Northumberland said that the presence of so many persons showed the interest taken by them in the hospital, and also that they were probably acquainted with its career. Perhaps, on this account, he ought not to detain the meeting long, but as he had the pleasure of being present, he could not help looking back to the time when, nearly 25 years ago, the hospital was first opened in Fitzroy Square. At that time the promoters were not absolutely sure whether their theory was a sound one, nor whether the venture would be a success. Their first efforts to found a pay hospital had failed; they were not allowed to open it, and were left with the house on their hands. This was a very great discouragement, and it was due to the zeal and determination of those concerned in the undertaking, and most of all to Sir Henry Burdett, Mr. Cox, and others who had the scheme so much at heart, that, undismayed by their disagreeable experience and all the difficulties of the situation, they persevered until this establishment was opened and successfully worked. He would like to remind his hearers that the hospital was started on two assumptions, the first of which was that it was a good thing that people should, as far as they were able, pay for the benefits of hospital treatment. That, he thought, was a broad principle, which was, and had been in the past, very usefully inculcated; it was the inculcating of that principle which had led, in some instances, to the establishment of pay wards in the hospitals; and in these days, when the doctrine that it was very comfortable to get other people to pay for advantages which one enjoyed was particularly prevalent, it was a good thing to set an example of paying, as far as one could, for the benefits enjoyed in hospitals and other places for the treatment of disease. There was another assumption, and it was this, that there was a class who, either because they could not afford it, or because they had not at home the facilities for proper treatment in illness, were not able to obtain advantages which they might otherwise have, but who were, and very properly, unwilling to take advantage of the free hospitals in this country. And it was thought that if an institution where they could be properly treated at an outlay which they could meet were started, they would gladly embrace the opportunity. It was not entirely a case of pounds, shillings, and pence. There were cases where, either because people lived in the country, or because a house which might be efficient enough for other purposes was not efficient enough to supply the inmates with the comforts required in illness, it was a great advantage to obtain those comforts in an institution like this. The proof of the pudding was, it was said, in the eating, and the experience of 25 years had shown that the founders were right, and that there was a class which gladly embraced the opportunity offered, and would no doubt continue to do so.

### An Up-to-date Hospital.

People might say, Why are you not contented with what you have done, and why do you come before the public asking for funds? He had great sympathy with that feeling, and if it had been merely a question of enlarging this institution he might not perhaps have agreed to the appeal being made, although it might be thought by those who had watched over the institution all this time that it should fall to the lot of others to provide further accommodation. That, however, was not quite the difficulty. It was entirely owing to the medical and surgical profession. They would go on discovering fresh microbes and inventing fresh ways of destroying them. They were for ever inventing fresh means of dealing with disease, and could now take one to pieces, as a child did with a puzzle, and put one together again, and think nothing of it. Appliances which were considered ample 25 years ago were not so now; and if an institution of this kind was to exist, it could only do so by guaranteeing that patients should be treated in such a way that they should have all the advantages of modern science, and be protected from the dangers of surgical operations by having all the new methods of treatment assured to them. When it was pointed out to him that this house was rather behind the day, he felt that the suggested change must be made so as to bring it up to date. He was very glad that as a secondary matter it had been found possible to add to the accommodation; that, however, was not the first consideration, and the committee had done all they could to put at the disposal of those who tended the patients the full means of carrying out their work to the very best advantage, so as to secure, as far as possible, immunity from those dangers which sometimes attended operations. It was the very greatest gratification to him to take part in this very important further development. It would be a still happier day when the committee could say they wanted no more money, and were laying up that sinking fund to which Sir Henry Burdett had alluded.

### Progress in Medicine and Surgery.

Sir William Church, in seconding, said there could be no doubt that many practising doctors had had occasion to feel grateful for the existence of this hospital, and for certain changes in other hospitals, consequent upon this house having shown the way. Fitzroy House was excellent in many ways as it was, but he thought all the members of his profession would most cogently endorse what his Grace the Duke of Northumberland had said as to the necessity for remodelling it in accordance with new requirements. The 22 years that had elapsed since it was built had been perhaps the most important in surgery and medicine since the world had had a history; the knowledge of the progress of disease had enormously increased, and Fitzroy House would not have been fulfilling its duty if it had not placed all possible means of treatment at the service of the patients. This increase of knowledge was more apparent in the surgical than in the medical branches of science, since in the latter there was not so striking an effect, and for this reason it was sometimes thought that surgery had increased in the benefits it was able to give to mankind to a greater extent than medicine had. Ideas of disease had entirely altered. He had no doubt that if any of those present were alive in another 22 years, other changes would be made, and remedies discovered which in some cases at any rate would actually extinguish disease. He was sure that all consulting practitioners in London fully realised the immense benefit this hospital had been to their patients, and to themselves in greatly alleviating their labours. He most heartily joined with his Grace in placing the resolution before his hearers.

The resolution having been carried, a vote of thanks to the Bishop of Southwark was proposed by Mr. F. D. Mocatta. He assured the Bishop that his presence gave great support to the cause that was in hand. He himself was one who recollected the opening of that hospital, and he had always been conscious of the need for such an institution. It was impossible for people of moderate means to afford the best medical and surgical advice without some such institution. Speaking of the nursing, Mr. Mocatta remarked that the generation of nurses who ruled in his young days had given place to one of a very different stamp. Things that were possible now, both in medicine and surgery, would hardly have been credited 20 years ago. Such a home as this, in which people of moderate means might enjoy the blessings of science, was a great boon, and insured every hope of restoration to health in such cheerful surroundings. He had a horror of gratuitous charity, and believed that that form of charity which was remunerative was the best; it was more likely to do good when it did not necessitate a feeling of dependence. He thanked the Bishop very much for the encouragement derived from his presence among them.

Mr. Christopher Heath seconded the motion. He wished more of the clerical profession had been there.

### The Self-respecting Workman.

The Bishop of Southwark said the services he had rendered were so minute that he thought the speeches must have been made in order to enable him to propose the next resolution, viz., a vote of thanks to the Duke of Northumberland for presiding. Evidently the Duke had the quality of sticking to the thing he put his hand to, since it was 20 years since he was present at the opening of that hospital, and he had been faithful to it all through.

### Honour where Honour is Due.

Sir Henry Burdett, in seconding the resolution, said: With regard to this pay hospital, which those present were cordially invited to inspect, the promoters were very much indebted to certain people who had worked very hard to get it ready in time for the opening in the beginning of January. He would especially mention the architects, Messrs. Young and Hall; the builders, Messrs. Prestige and Co., who had shown great personal interest in the work; and the foreman painter, Mr. Rogers, who had been on duty hour after hour, anxious that the work should be well done. He thought people who did really good work should have their due meed of praise, and it was only just to mention these firms, with Mr. Stallibras, of Messrs. Maple and Co. who supplied the furniture, and had done yeoman service in many ways.

### The President.

The president, the Duke of Northumberland, was one of those supporters of the hospital who should have special thanks. As Earl Percy he represented his father at the opening in 1880, and he had helped the hospital to a very great extent. Indeed, but for his help this opening ceremony would not have been possible. The Duke wished him not to say what the circumstances were, but they might take it from him that the house would not have been rebuilt but for the Duke of Northumberland. He himself, as well as Mr. Cox, had great doubts about the hospital's future, and if the Duke had not come forward Fitzroy House might have been closed for ever. Nothing that he could say would more commend the vote to the meeting. It was well known that if the Northumberlands put their hand to any object they were a tower of strength—certainly they had been so to this hospital.

### The Lady Superintendent.

Their indebtedness to Miss Pearson must not be forgotten. She had had a most harassing time. Every lady knew what it was to have workmen in the house, and to be trying to get them out. Last Sunday he did not think it would be possible to be ready in time, but it had been done, and Miss Pearson and the housekeeper, by sheer unselfish devotion, had swept the workmen out. They deserved their most hearty thanks. The vote of thanks to the president was carried with acclamation.

The Duke said he was extremely obliged for the expression of the vote, and for the way in which it had been received. He did not think Sir Henry Burdett was quite correct in saying that this opening would not have taken place but for his help; he was quite sure that the interest Sir Henry himself took in it would not have allowed him to rest content without achieving the desired result; by hook or by crook he would have accomplished it. Knowing his character, he did not believe he would have been disheartened—"he would have got it done somehow." But it was quite true that he (the speaker) took a great interest in the hospital, and he was hopeful enough to believe that work begun under such auspices must succeed. There was one duty which he would ask to be allowed to perform: Sir Henry Burdett was the founder, in a great measure, of this movement; Miss Pearson had conducted her work with the greatest energy and tact; and Mr. Cox had from the very earliest times taken a most onerous and active part. Indeed, the whole committee had worked with very great zeal and attention, and he would suggest that a special vote of thanks be given to those he had mentioned.

Mr. Bland Sutton seconded this. It was unnecessary, he said, to say anything about the work of Sir Henry Burdett, because those who were present knew him as well as he himself did; they knew also his great work, and how much this institution owed to him. With regard to Mr. Cox, though aware of his great interest in Fitzroy House, it was not until he was himself asked to serve on the committee that he realised what that meant, and how important Mr. Cox was in the domestic concerns of the house. Some young men looked upon business as irksome, and Mr. Cox's attention and dispatch were a lesson that all might profit by. He did not know how to say enough of Miss Pearson, whom he had known for many years. She was an admirable nurse, and could assist at a major operation as well as a surgeon. Latterly she had proved herself a veritable clerk of the works; she had excellent taste in furniture, understood paint, and was a wonderful judge of colour. As long as she was in office he was sure the hospital would flourish.

The resolution having been carried with enthusiasm, the meeting was over, and the house, described in last week's Hospital, was inspected.

### The Firms Employed.

Appended is a full list of the firms engaged:—

The Architects.—Messrs. Young and Hall, 17 Southampton Street, Bloomsbury, W.C.

General Contractors.—Messrs. Prestige and Co., 149 Grosvenor Road, S.W.

Hot Water Engineering and Cooking Apparatus.—Messrs. James Slater and Co, 251 High Holborn, W.C.

Sanitary Fittings and Plumbing.—Messrs. Dent and Hellyer, 21 Newcastle Street, Strand.

Electric Lighting, Fittings, and Power Work; also Bells and Telephones.—Messrs. Duncan, Watson and Co., 100 Charing Cross Road, W.C.

The whole of the Fireplaces have been supplied by the Teale Fireplace Company, 28 Berners Street, W.

The Electric Lift.—By the B. and S. Folding Gate Company, 19 Tower Street, Upper St. Martin's Lane, W.C.

The Papyrolith Flooring was laid by the Papyrolith Company, Dashwood House, New Broad Street, E.C.

The Enamel Paint work generally is Paripan, made by Messrs. Randall Brothers, Palmerston Buildings, E.C.

The Opalite has been carried out by the National Opalite Glazed Brick and Tile Syndicate, 39 Hamilton House, Bishopsgate Street.

The Plastering has been made by the Granite Silicon Plaster Company, Camden Town.

Furniture.—Maple and Co., Limited.

